# Evaluation of reference-based two-color methods for measurement of gene expression ratios using spotted cDNA microarrays

**DOI:** 10.1186/1471-2164-7-35

**Published:** 2006-02-24

**Authors:** Bernardo R Peixoto, Ricardo ZN Vêncio, Camila M Egidio, Luisa Mota-Vieira, Sergio Verjovski-Almeida, Eduardo M Reis

**Affiliations:** 1 Departamento de Matemática, Universidade dos Açores, 9501-801 Ponta Delgada, Açores, Portugal.; 2 BIOINFO-USP, Núcleo de Pesquisa em Bioinformática, Instituto de Matemática e Estatística, Universidade de São Paulo, 05508-090 São Paulo, SP, Brasil.; 3 Departamento de Bioquímica, Instituto de Química, Universidade de São Paulo, 05508-900 São Paulo, SP, Brasil.; 4 Unidade de Genética e Patologia Moleculares, Hospital do Divino Espírito Santo, 9500-370 Ponta Delgada, Açores, Portugal.

## Abstract

**Background:**

Spotted cDNA microarrays generally employ co-hybridization of fluorescently-labeled RNA targets to produce gene expression ratios for subsequent analysis. Direct comparison of two RNA samples in the same microarray provides the highest level of accuracy; however, due to the number of combinatorial pair-wise comparisons, the direct method is impractical for studies including large number of individual samples (e.g., tumor classification studies). For such studies, indirect comparisons using a common reference standard have been the preferred method. Here we evaluated the precision and accuracy of reconstructed ratios from three indirect methods relative to ratios obtained from direct hybridizations, herein considered as the gold-standard.

**Results:**

We performed hybridizations using a fixed amount of Cy3-labeled reference oligonucleotide (RefOligo) against distinct Cy5-labeled targets from prostate, breast and kidney tumor samples. Reconstructed ratios between all tissue pairs were derived from ratios between each tissue sample and RefOligo. Reconstructed ratios were compared to (i) ratios obtained in parallel from direct pair-wise hybridizations of tissue samples, and to (ii) reconstructed ratios derived from hybridization of each tissue against a reference RNA pool (RefPool). To evaluate the effect of the external references, reconstructed ratios were also calculated directly from intensity values of single-channel (One-Color) measurements derived from tissue sample data collected in the RefOligo experiments. We show that the average coefficient of variation of ratios between intra- and inter-slide replicates derived from RefOligo, RefPool and One-Color were similar and 2 to 4-fold higher than ratios obtained in direct hybridizations. Correlation coefficients calculated for all three tissue comparisons were also similar. In addition, the performance of all indirect methods in terms of their robustness to identify genes deemed as differentially expressed based on direct hybridizations, as well as false-positive and false-negative rates, were found to be comparable.

**Conclusion:**

RefOligo produces ratios as precise and accurate as ratios reconstructed from a RNA pool, thus representing a reliable alternative in reference-based hybridization experiments. In addition, One-Color measurements alone can reconstruct expression ratios without loss in precision or accuracy. We conclude that both methods are adequate options in large-scale projects where the amount of a common reference RNA pool is usually restrictive.

## Background

Gene expression studies using either oligonucleotides or spotted cDNA microarray platforms are based mainly on data generated in single or dual-channel analysis. While industrially manufactured oligonucleotide arrays (e.g. Agilent Whole Genome 44 k Oligoarray and CodeLink Whole Genome Bioarray) have optimized protocols that perform well on both single and dual-channel microarray experimental designs, custom made spotted cDNA or oligonucleotide microarrays have been mainly employed in two-channel designs [1]. Single-channel (one-color) experiments probe one RNA sample per hybridization, whereas dual-channel (two-color) experiments generate spot signal intensity values from two different RNA samples, each labeled with one of two cyanine dyes (Cy3 or Cy5), followed by simultaneous hybridization. After subtraction of background signal, absolute intensity values, derived from each channel, are often used to calculate expression ratios for subsequent analysis. The ratio of the signal intensities obtained from the two channels is a relative measure of gene expression of the corresponding gene probe. Ratiometric data analysis minimizes various sources of variation related to the construction and hybridization of the microarrays, thus providing the highest level of precision in the comparison of gene expression profiles from two different RNA samples [2].

While direct hybridization of two experimental RNA samples in the same slide is highly desirable, indirect comparison through the co-hybridization of a test sample together with a common reference standard is the most used experimental design [3,4]. Relating each experimental sample to a common reference standard facilitates the comparison of ratios across datasets [4]. Several types of reference samples based on commercial Universal reference RNA [5], genomic DNA [6-8] or PCR products representing the collection of cDNA clones printed on the chip [9,10] have been proposed, but no single universal reference standard is widely adopted, seriously impeding cross comparisons between different studies. The composition and properties of the selected reference sample must be addressed properly, because it raises issues concerning the experimental design, the goal of the study and the long term comparability of the data. For example, a problem associated to the use of a reference RNA in tumor profiling studies is the requirement of a large amount of high-quality reference sample to allow comparison across multiple datasets [11-13]. Pooling of equal amounts of RNA from test tumor samples is impractical in prospective studies, because samples collected after the construction of the pool would not be represented, precluding adequate comparison of recently collected samples. Cell lines may in principle be an unlimited source of a reference RNA. Indeed several tumor profiling studies have employed such a method [11,12,14]. However, the biological variability inherent to cells cultivated in different batches requires that all RNA used to generate the reference pool be prepared prior to the beginning of the hybridizations. As an alternative to reference-based designs, theoretical and experimental work have shown that other types of two-channel designs, namely loop-designs, may produce precise estimates of differential gene expression compared to a design based on a common RNA reference [15]. However, Dobbin and Simon [16] have demonstrated that for experiments aiming to discover clusters within a collection of samples (class discovery), a common goal of cancer profiling studies, the reference design is more robust than the loop design. According to these authors, variable quality of individual arrays may have a greater impact on cluster analysis when a loop design is used. This consideration is particularly significant in studies using in-house spotted-cDNA microarrays, in which uneven quality between slides of different batches may limit the loop approach.

In this study we designed a set of experiments to evaluate the precision and accuracy of gene expression ratios derived from two-color microarray hybridizations using each of three tumor tissue RNA samples and two different external references: (i) a pooled tumor RNA sample (RefPool) that was labeled in parallel with the test tissue sample; and (ii) a 27-mer reference oligonucleotide (RefOligo) complementary to every feature of the array which was labeled by chemically coupling of a fluorescent nucleotide. The RefOligo method was originally proposed by Dudley et al. to control intensity ratios of gene expression studies in yeast [17], a system with much lower gene expression complexity. Here, ratios derived from the direct pair-wise hybridizations of human tissue samples were taken as the gold-standard against which ratios derived from reconstructed measurements were compared. These external-reference based ratios were also compared to ratios reconstructed from one-color measurements. The results are discussed based on the strengths and weaknesses of each of the three indirect experimental designs.

## Results and discussion

### Experimental design

**Figure 1:**
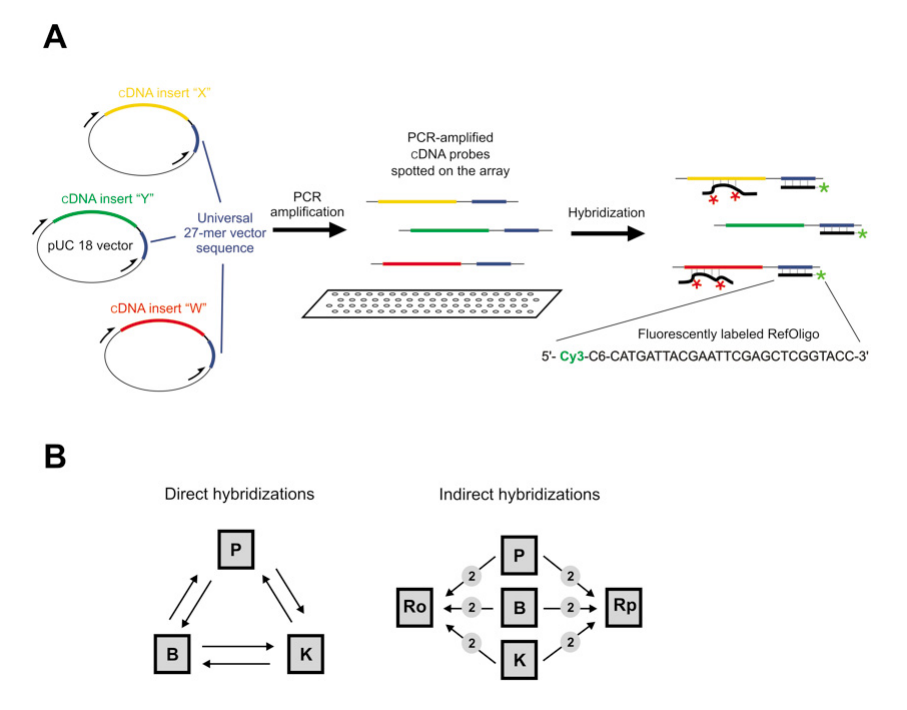
**Schematic representation of the experimental design. **(A) Human cDNA inserts that were cloned into pUC18 vector were amplified by PCR with vector universal primers in such a way that each PCR-amplified cDNA probe spotted on the array contained a common 5'-end sequence derived from the vector. The RefOligo consisted of a synthetic Cy3-labeled 27-mer oligonucleotide (marked with green asterisks) that is complementary to the 5'-end vector sequence present in each cDNA probe. This RefOligo common reference was co-hybridized to the arrays along with Cy5-labeled tissue-derived targets (marked with red asterisks). (B) Overview of the experimental design including direct and indirect hybridizations. Filled squares represent fluorescent targets derived either from test RNAs (B, Breast; K, Kidney; P, Prostate), or from two types of external references, RefPool (Rp) or RefOligo (Ro). Arrows indicate co-hybridized target RNAs -- arrow head indicate the target labeled with Cy3, whereas the arrow tail indicate the target labeled with Cy5. The number of replicate hybridizations performed with each paired sample is indicated in the small circles.

RNA isolated from three types of human tumor tissues (prostate, breast and kidney) was used to generate fluorescent targets for microarray hybridizations. Finding differentially expressed genes among different cancer tissues and cell lines and identifying gene expression signatures for each of them is a frequent task in the study of human gene expression using microarrays [11,18,19]. In addition, comparative gene expression profiling among tumors derived from different organs are revealing common gene signatures with highly significant correlation to clinical behavior of the cancer [20]. Tissue-derived fluorescent targets were co-hybridized with each of two different types of external references; in one set of experiments, a fixed amount of a 27-mer 5'-end Cy3-labeled synthetic oligonucleotide (RefOligo; Figure 1A) was co-hybridized with Cy5-labeled cDNA targets derived from RNA of each tissue (Figure 1B). In parallel, Cy5-labeled cDNA targets derived from each tissue were co-hybridized to Cy3-labeled targets from a reference RNA pool (RefPool) (Figure 1B). RefPool was assembled from equimolar amounts of RNA isolated from each of the three tumor tissues. Two replicate hybridizations were performed for each tissue, using either RefOligo or RefPool as an external reference (Figure 1B – indirect hybridizations). In turn, each replicate slide had two replicate cDNA arrays spotted on them, thus generating four intensity measurements for each experiment. In addition, RNA aliquots from each tissue were labeled with either Cy3 or Cy5 and hybridized directly to each other. A replicate hybridization with dye-reversal was performed for each pair of tissues (Figure 1B – direct hybridizations), again generating four intensity measurements. Gene expression log-ratios between each pair of tissues were reconstructed using either RefOligo or RefPool reference values and compared to ratios obtained from direct hybridizations, which we considered herein as the gold-standard. To evaluate the effect of the external references, we compared RefOligo and RefPool ratios to ratios reconstructed from single-channel measurements (One-Color). One-Color ratios were calculated directly from intensity measurements of Cy5-labeled targets from the tissue samples that were co-hybridized in RefOligo experiments. It has been proposed that intensity measurements obtained from spotted oligonucleotide arrays are not affected by co-hybridized targets [21]. Nonetheless, we opted to obtaining One-Color intensity measurement from RefOligo hybridizations, instead of from RefPool, to ensure that Cy5 intensity values would not be affected by targets labeled with Cy3 that would compete for the same probe sequence in the array.

**Figure 2:**
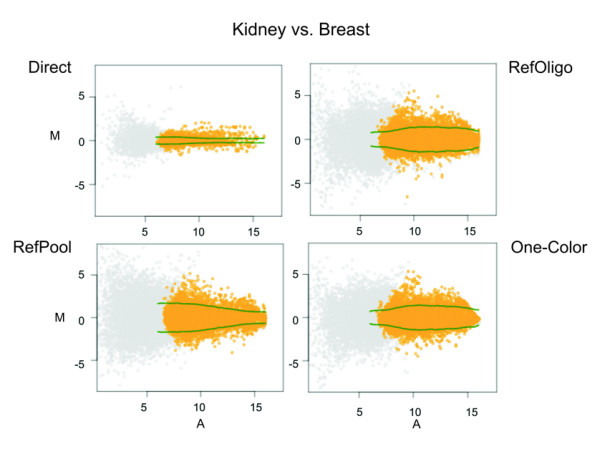
**MA–scatterplots for direct and reconstructed ratios. **Normalized intensity ratios for Kidney vs. Breast comparison from all technical replicates is shown. The yellow dots display the log_2 _intensity ratio (M) as a function of the log_2 _intensity product (A) for each gene on the array. Spots that were excluded from further analyses (with intensities below the local background plus 2 SD) are shown in gray. Green lines show the credibility interval thresholds (see Methods) used for estimation of differentially expressed genes.

A detailed description of data normalization, filtering and averaging of replicates is described in the Methods section. Intensity ratios for Kidney vs. Breast comparison are depicted in Figure 2 as MA-plots. It can be seen that ratios resulting from all three reconstructed methods, i.e. RefOligo, RefPool and One-Color analyses, are more dispersed than those from the direct comparison. Similar results were obtained for the other two tissue comparisons [see Additional file 1]. Correlation of log-ratios between the two forward and two reverse labeled replicates for each direct comparison was determined, and a high degree of correlation was observed (Kidney vs. Breast 0.937; Prostate vs. Breast 0.872; Prostate vs. Kidney 0.945). This result suggests that there is no significant residual bias associated with the use of two different fluorophores (Cy3 or Cy5) in the direct comparisons that might have precluded the use of only Cy5-labeled tissue samples for indirect comparisons, where we always labeled the reference with Cy3. Nonetheless, we can not rule out the possibility that the use of a dye-swap design in the direct hybridizations may have caused some compression in the direct ratios as compared to the reconstructed ones. All spots detected only in one channel (the other channel intensity being below the background) were excluded from further analyses. Total number of valid gene expression ratios is shown in Table 1. When an intensity-based filter cutoff of 2 standard deviations (SD) above local background was applied, a comparable fraction of ratios (~30 %) were selected in each reconstructed method, whereas in direct hybridizations only 20 % of the ratios were selected (Table 1). On average, arrays hybridized with Cy5-labeled targets derived from individual tissues showed lower non-specific background and produced a slightly larger fraction of valid intensity measurements when compared to Cy3-labeled targets derived from the same RNA samples (data not shown). This would explain the lower fraction of valid ratios in the direct comparisons, where a number of transcripts having an expression level near detection cutoff in one tissue would eliminate those genes from the analysis when labeled with Cy3. On the other hand, when Cy3-labeled tissue-derived targets were used as reference in the RefPool comparisons, this effect would not prevail in all situations because the abundance of low-expression messages could be compensated by its eventual higher expression in one of the other two tissues represented in the three-tissue pool. The homogeneous hybridization to all probes in the array obtained with the synthetic reference oligonucleotide would have a similar compensatory effect in the RefOligo analyses.

**Table 1:** Ratios from direct and indirect hybridizations. Total number of gene expression ratios calculated from all spots (All) or spots 2 standard deviations (2 SD) above the average slide background are shown. Percentages of ratios above 2 SD are shown in parenthesis.

	Kidney vs. Breast		Prostate vs. Breast		Prostate vs. Kidney	
	
	All	2 SD	All	2 SD	All	2 SD
RefOligo	3,541	1,044 (29%)	3,384	1,158 (34%)	3,406	1,012 (30%)
RefPool	3,447	891 (26%)	2,673	906 (34%)	2,678	820 (31%)
One-Color	3,542	1,035 (29%)	3,386	1,159 (34%)	3,407	1,010 (30%)
Direct	3,206	716 (22%)	3,124	706 (23%)	3,079	483 (16%)

### Performance among indirect hybridization methods

**Figure 3:**
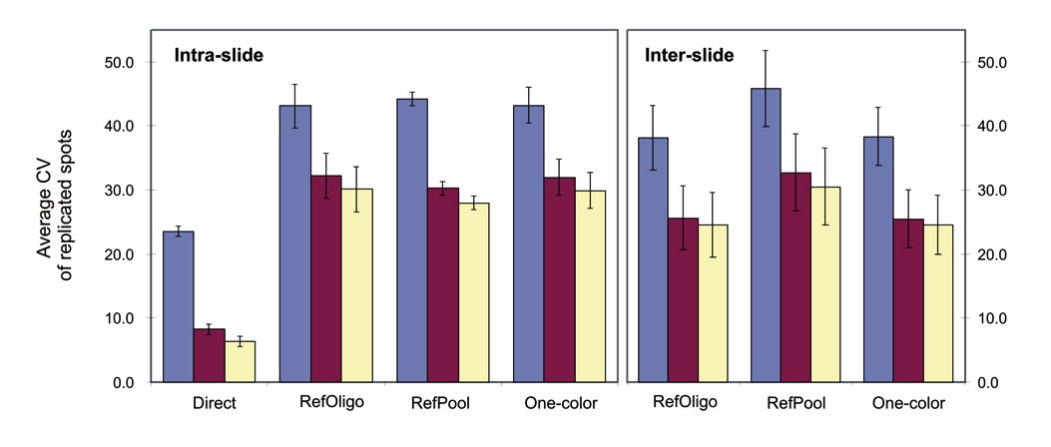
**Precision of direct versus reconstructed ratios as determined by the average coefficient of variance (CV) of intra- and inter-slide replicates. **Average CV of expression ratios derived from intra-slide spot replicates (left panel) or inter-slides (right panel) obtained in direct hybridizations or in indirect hybridizations (RefOligo, RefPool, One-Color). CVs were calculated using all ratios (blue bars), ratios calculated from spots that were one (red bars) or two (yellow bars) standard deviations above the local background. Standard errors from pair-wise comparisons of three tissues (Prostate vs. Breast, Prostate vs. Kidney or Breast vs. Kidney) are shown.

**Figure 4:**
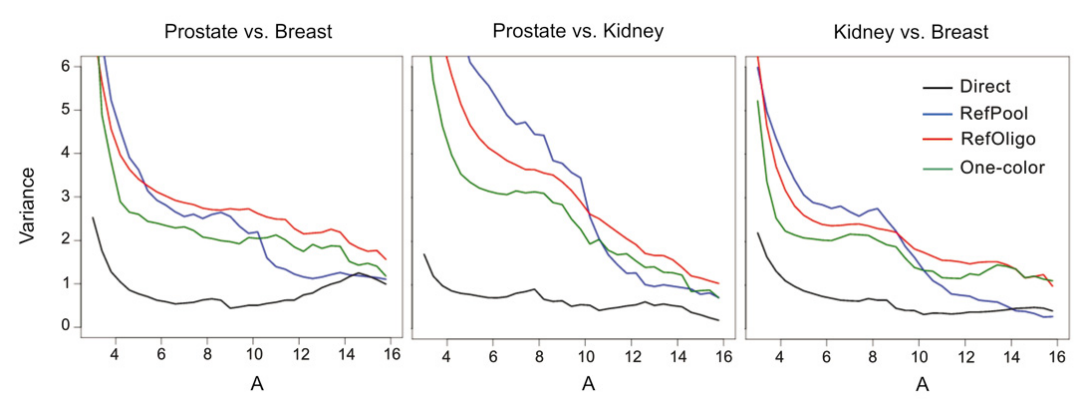
**Variance of reconstructed expression ratios along the range of expression intensities. **Each panel shows a different pair-wise tissue comparison. The graphs show the variance of log_2_-ratios (Y axis) from all technical replicates that was calculated by using a sliding window of 1.5, moving 0.4 at each step along the range of average expression log_2_-intensities A (X axis).

To estimate the precision of RefOligo and RefPool in reconstructing ratios obtained in direct hybridizations we calculated the coefficient of variation (CV) for intra- slide replicates (Figure 3, left panel). We found that when all ratios are used, the average CV of ratios reconstructed from indirect hybridizations are comparable (RefOligo, 43.1; RefPool, 44.2) and ~2-fold higher than that obtained in direct hybridizations (Direct, 23.6; Figure 3, left panel, blue bars). This result is in line with the data from Dudley and colleagues (2002), which showed that the average CV of replicate ratios reconstructed from a reference oligonucleotide is about twice that for direct ratios [17]. Ratios reconstructed from One-Color measurements showed CV values comparable to reference designs (One-Color, 43.2). When we applied intensity filters to select only spots 1 or 2 SD above the local slide background, we observed a consistent decrease in the average CV for both direct and reconstructed ratios (Figure 3, left panel, red and yellow bars). While CVs from direct ratios decreased up to 50 %, CVs from reconstructed ratios decreased approximately 30 % (Figure 3, left panel). Average CV for inter-slide replicates was also comparable for all reconstructed ratios (RefOligo, 38.1; RefPool, 45.8; One-Color, 38.4; Figure 3, right panel). Noteworthy, we observed no difference between the averages of intra- and inter-slide CVs (Figure 3). As all hybridizations were performed using slides from the same batch and were executed in parallel, we think that this may explain the similar values of CV among intra- and inter-slide replicates. The high correlations of raw intensities that we observed between intra- (average of 0.945 ± 0.019) or inter-slide spot replicates (average of 0.934 ± 0.068) across all tissue comparisons support this notion. Taken together, these results indicate that the precision of all indirect measurements are comparable and that One-Color measurements are sufficient to reconstruct ratios as precise as those obtained from external references. Precision of reconstructed ratios was also evaluated in terms of the average variance of all valid expression ratios measured across the range of expression intensities (Figure 4). While all indirect measurements showed similar profiles, it is apparent that variances of reconstructed ratios are higher than that observed for direct ratios, particularly in the low intensity range (Figure 4). We also observed that variances from One-Color ratios show a similar profile of variation as that obtained from RefOligo and RefPool (Figure 4).

**Figure 5:**
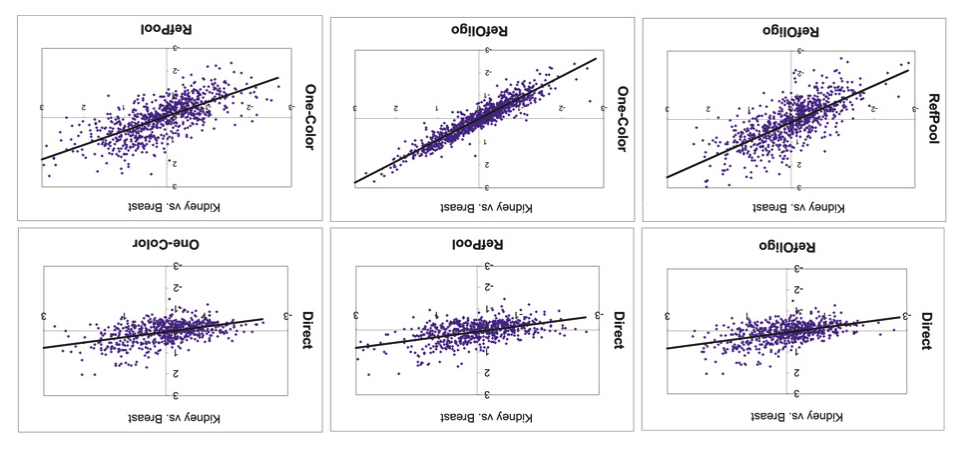
**Correlation between direct and reconstructed ratios. **Ratio plots from Kidney vs. Breast comparisons are shown. Upper panels: scatter plots between direct ratios and each of the three reconstructed ratios (RefOligo, RefPool, One-Color). Lower panels: scatter plots between the reconstructed ratios. Ratio plots for the remaining tissue comparisons are shown as supplementary material [see Additional file 2]. In all cases, only ratios from spots with intensity values 2 SD above the average slide background were used.

The accuracy of indirect methods was estimated by correlation analysis between direct ratios and reconstructed ratios. Representative scatter plots between direct and reconstructed ratios are shown in Figure 5. Pearson's correlation among different tissue comparisons ranged from 0.26 to 0.56 and 0.31 to 0.58 for log-ratios reconstructed from RefOligo and RefPool, respectively (Table 2 – Pearson). A similar range of Pearson correlation values was obtained for log-ratios from One-Color measurements (0.28 to 0. 53). All Pearson correlations were statistically significant (p-value for rejecting Ho: r = 0 near zero). Very similar results were obtained by using non-parametric Spearman's rank-correlation (Table 2 – Spearman). This result indicates that the RefOligo approach produces expression measurements as accurate as those obtained with a common RNA reference. It also provides evidence that gene expression ratios as accurate as those obtained from reference-based designs can be reconstructed from One-Color measurements collected from different slides.

**Table 2:** Correlation coefficient analysis between direct and reconstructed expression ratios. Only ratios from spots with intensity values 2 SD above the average slide background were used to calculate Pearson's correlations or Spearman's rank-correlations. The number of intensity values used in each correlation analysis is shown (n). All Pearson correlation values were statistically significant (p-value for rejecting Ho: r = 0 << 10^-5^).

	Kidney vs. Breast			Prostate vs. Breast			Prostate vs. Kidney		
	Pearson	Spearman	n	Pearson	Spearman	n	Pearson	Spearman	n

Direct vs. RefOligo	0.47	0.45	672	0.56	0.53	671	0.26	0.22	464
Direct vs. RefPool	0.48	0.43	643	0.58	0.57	632	0.31	0.19	445
Direct vs. One-Color	0.45	0.42	669	0.53	0.49	671	0.28	0.25	464

### Precision of indirect hybridization methods to reconstruct direct measurements

**Figure 6:**
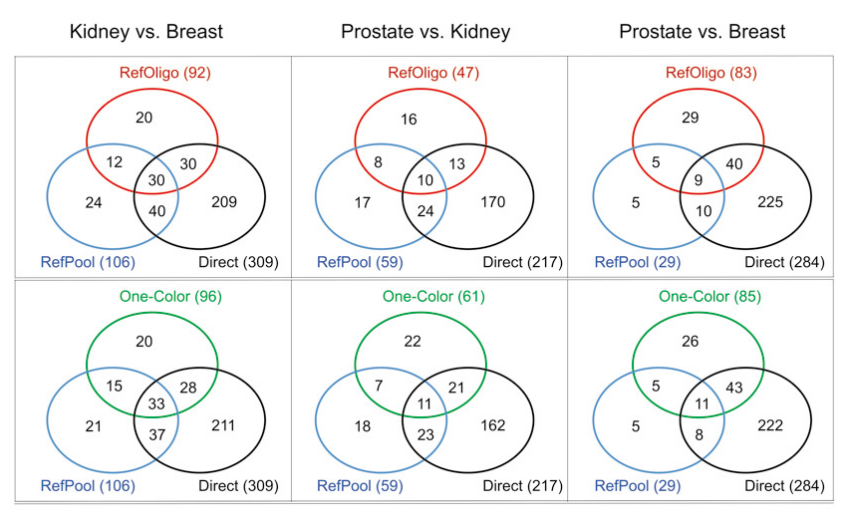
**Comparison of genes identified as differentially expressed in direct and reconstructed ratios. **Each of the three pair-wise tissue comparisons is shown at the left, center or right panels as indicated at the top of the figure. Genes identified as differentially expressed by direct and indirect methods were selected based on 95 % credibility intervals (see Methods). Upper panels: genes identified by the RefOligo analysis are compared to those identified in RefPool and Direct analyses, and displayed as unions and intersections using Venn diagrams. Lower panels: genes identified by the One-Color analysis are compared. The total number of genes identified by each method is indicated in parenthesis.

The ability of each indirect method to identify the same set of genes found in the direct comparisons was evaluated. Genes differentially expressed in the direct hybridizations between each pair of tumor tissues were selected using 95 % credibility intervals for differential expression [22], and were taken as the standard gene set (see Methods for details). Concordance and discordance among gene sets identified by direct and reconstructed ratios were represented as Venn diagrams (Figure 6). On average, a similar fraction of concordant genes, i.e., genes that were also identified in the correspondent direct hybridization, was identified as differentially expressed by each indirect method (RefOligo: average of 58 % among the three pair-wise tissue comparisons; RefPool: average of 63 %; One-Color: average of 60 %). It is noteworthy that all three indirect methods detected a small fraction (15 to 18 %) of the standard set of differentially expressed genes. Also, RefOligo, RefPool and One-Color identified similar fractions of false-positive genes, i.e. genes present in reconstructed ratios but not in the standard set (RefOligo: average of 42 %; RefPool: average of 37 %; One-Color: average of 40 %). To document whether the high rate of false positives in the RefOligo analysis was due to conditions inherent to our system or is a general feature of the RefOligo method, we estimated the number of false positives using data produced by Dudley et al. (2002). As it was not possible to estimate credibility intervals based on self-self ratios, in this case we used a 2-fold change threshold to select genes differentially expressed between yeast cells grown either in glucose or galactose. Using this criterion, we found that approximately 62 % of the genes identified as differentially expressed by ratios reconstructed from a reference oligonucleotide in that work were false-positives (data not shown). The high number of false-positives identified by reconstructed ratios may, in part, be explained by the limited number of technical replicates tested in the present work. We speculate that the inclusion of more technical replicates, or even biological replicates, may contribute to decrease the number of false-positive genes. In spite of the limitations pointed above, spotted cDNA microarray analysis is a powerful tool to identify candidate genes in comparative gene expression studies in cancer. Validation of differential expression by independent methods such as quantitative RT-PCR will remain as a necessary further step to confirm them as true tumor-associated molecular markers.

## Conclusion

Data presented here show that RefOligo is a reliable alternative to a RNA pool in reference-based hybridization experiments when studying organisms with a complex transcriptome such as humans. The implication of our results is that the unlimited availability of an inexpensive (~US$ 0.30 per hybridization), chemically synthesized reference oligonucleotide makes its use very convenient in large-scale projects where the availability of an RNA pool is usually restrictive. The RefOligo method enables an efficient and flexible experimental design because one may relate expression measurements to a common reference (dual-channel) or, alternatively, use intensity values only (single-channel) [17]. As the RefOligo is complementary to every array element, fluorescent signal derived from bound RefOligo can be used to assess spot quality and facilitates array griddling by image processing software. We speculate that RefOligo will improve comparison of data obtained from different batches of spotted arrays, by correcting for small variations in spot morphology and in the amount of spotted DNA across batches. Moreover, signal intensities of bound reference oligonucleotide molecules correlate well with the amount of cDNA probes present in each spot [23], suggesting that expression ratios reconstructed from RefOligo may accurately reflect the absolute abundance of each transcript present in the RNA population [17].

Another important conclusion of our study is that use of One-Color ratios does not compromise precision more than other currently used methods based on indirect measurements. This observation is in line with recent evidence showing that gene classifiers based on intensity measurements may outperform ratio-based classifiers [24]. Intra- and inter-slide expression intensity values obtained with One-Color correlates well and reveal acceptable number of false positives as compared to reference-based methods. Given that direct measurements in datasets containing large number of individual samples (e.g., tumor classification studies) is impractical, One-Color based analysis allows direct comparison of measurements across all samples, with a considerable reduction in costs since it eliminates the requirement of labeling a reference sample

## Methods

### Tissue samples

Prostate, kidney and breast tumor samples were obtained from freshly-frozen tissue collections maintained by Instituto Nacional de Câncer, Rio de Janeiro (prostate adenocarcinomas, renal cell carcinomas) and Unidade de Genética e Patologia Moleculares, Hospital do Divino Espírito Santo, Portugal (breast adenocarcinomas). All samples were collected between 1999 and 2002 with informed consent from patients submitted to surgery, and were snap-frozen in liquid nitrogen within 10 min from resection. All samples were examined by a pathologist at each Institution, and, in the case of prostate, hematoxylin/eosin stained micro-sections obtained from each side of the frozen blocks were used to grossly delimit the spatial distribution of the tumor mass. If necessary, tissue blocks were further dissected to warrant that at least 70% of the section used for RNA extraction was composed of malignant cells. Macro-dissected tumor samples were returned to liquid nitrogen until use.

### RNA isolation

Total RNA was isolated with TRIzol (Invitrogene) using the protocol recommended by the manufacturer. For each tissue, aliquots of RNA isolated from 5 patient samples were pooled, in order to minimize the effect of biological diversity within each set of tissue samples, as well as to circumvent the problem of limited amounts of RNA available from each individual tumor sample. A reference RNA pool (RefPool) was assembled by combining equal amounts of RNA from each one of the three tumor tissues. The amount and quality of each RNA sample was verified on an Agilent 2100 Bioanalyser.

### Target labeling and hybridization

RNA samples were labeled with either Cy3- or Cy5-labeled nucleotides (CyScribe first-strand cDNA labeling kit, Amersham Biosciences, Piscataway, NJ), using 10 μg of DNAse-treated RNA and a mixture of oligo-dT and 9-mer random oligonucleotides.

For the indirect hybridizations, Cy5-labeled cDNA targets derived from RNA of each tissue were co-hybridized to either a fixed amount (100 pmol) of a 27-mer 5'-end Cy3-labeled reference oligonucleotide (RefOligo; 5'-CATGATTACGAATTCGAGCTCGGTACC-3', Sigma Aldrich Inc.), or to Cy3-labeled cDNA targets from the reference RNA pool (RefPool) labeled as described above with CyScribe first-strand cDNA labeling kit (Amersham Biosciences, Piscataway, NJ). The 27-mer RefOligo has no sequence similarity to any human expressed sequence available in GenBank. For RefOligo and RefPool, two replicate hybridizations were performed for each tissue. For direct hybridizations, fluorescently-labeled targets derived from each test tissue sample were combined pair-wise. A replicate hybridization with dye-reversal was performed for each pair of tissues.

Targets were evaporated in a Speedvac, resuspended in hybridization solution (50% formamide, 25% Amersham Microarray Hybridization Buffer V.2, 25% H_2_O) and manually hybridized for 16 h at 42°C to microarray slides containing 4,608 different cDNA probes, each spotted in duplicate on either half of the slide. A detailed description of the spotted cDNA microarray platform used is presented elsewhere [25]. Following hybridization, slides were washed (1.0× SSC, 0.2% SDS 10 min. at 55°C, 0.1× SSC, 0.2% SDS 10 min. at 55°C, 0.1× SSC, 0.2% SDS 10 min. at 55°C, 0.1× SSC 1 min. at RT, 0.1× SSC 1 min at RT, dH2O 10 sec. at RT) and dried with a N_2 _stream. Processed slides were scanned with a PMT setting of 700 V (GenIII Scanner – Amersham Biosciences) and background-subtracted artifact-removed median intensities of both Cy3 and Cy5 emissions were extracted for each spot from raw images using ArrayVision V.7.2 software (Imaging Research Inc., Ontario, Canada).

### Data normalization, filtering and averaging of replicates

To correct for systematic biases on the data originated from small differences in the labeling and/or detection efficiencies between the fluorescent dyes, both direct and reconstructed expression ratios were logged (base 2) and normalized using a locally weighted linear regression (LOWESS) algorithm [2,26] implemented as scripts written in R language [27]. Unless indicated, only spots whose background-subtracted intensities measured in both channels were 2 standard deviations above the local background (defined for each sub-array by a set of plant cDNA negative control probes) were considered in the analysis. For indirect ratio reconstructions, LOWESS normalization was performed in the M vs. A space, where:

${\text{M}}_{\text{rec}}=\log_{2}\left(\frac{\text{test sample 1 }(\text{cy5})/\text{reference }(\text{cy3})}{\text{test sample 2 }(\text{cy5})/\text{reference }(\text{cy3})}\right)$

and

A = log_2 _[test sample 1(cy5)]/2 + log_2 _[test sample 2(cy5)]/2

As there were 2 technical replicates for each indirect hybridization, and each cDNA probe was deposited in duplicate in each slide, 8 possible reconstructed expression ratio values could be generated for a given cDNA probe: K1L/B1L, K1R/B1R, K1L/B2L, K1R/B2R, K2L/B1L, K2R/B1R, K2L/B2L and K2R/B2R, where K and B denote Kidney and Breast for example, 1 and 2 denote different hybridizations and R and L denote the right and left spot sets from each slide, respectively. Final ratio values was obtained by taking the median value of all 8 reconstructed ratios for each cDNA probe, for RefOligo, RefPool and One-Color comparisons.

For direct hybridizations, LOWESS normalization was performed by combining log_2 _ratios from dye-swap replicate experiments as described in [26]. For direct hybridizations, LOWESS normalization was performed in the M vs. A space where:

M_dir _= 0.5 * log_2_(cy5/cy3 * cy3'/cy5') =

= 0.5 * log_2_(sample 1 (cy5)/sample2(cy3) * sample 1(cy3')/sample 2(cy5'))

and

A = 0.25 * log_2_(Samaple 1(cy3) * sample 1(cy5) * sample 2(cy3) * sample 2(cy5))

Raw and processed data files from direct and indirect hybridizations are available at author's website [28].

### Statistical analyses

Precision of intra-slide replicates from direct or reconstructed ratios was estimated by calculating, for each tissue comparison, the average coefficient of variance (CV) of the two replicated spots representing the same cDNA that are present in the microarrays. Final average intra-slide CV +/- SD from RefOligo or RefPool was calculated from the average intra-slide CV of each method measured in each tissue comparison. Average inter-slide CV was estimated by calculating the average CV of reconstructed ratios measured across the same spots in different slides.

To evaluate the variance of expression log_2_-ratios reconstructed from RefOligo or RefPool along the range of expression intensities, MA-plots were generated for each hybridization method (for each tissue comparison) using M and A values calculated from all replicates as defined above. For this analysis, the 2 SD intensity filter was not applied to access the variance on the entire range of intensities. Next, a sliding window of 1.5, moving 0.4 at each step, was used to calculate the variance of reconstructed ratios M along the range of expression intensities A (X axis). These analyses were performed using scripts written in R [29]. The R scripts used in all analyses described in the present work are available at author's website [28].

To evaluate the accuracy of expression ratios reconstructed from RefOligo, RefPool and One-Color measurements, a set of genes differentially expressed between each pair of tissues based on the direct hybridizations was identified, and was defined as the gold-standard set. Genes that were differentially expressed in the pair-wise tissue comparisons were selected with the statistical approach described in [22]. In short, the HTself method classifies an expression ratio as significant or not according to an experimentally derived intensity-dependent fold-change cutoff [22]. These cutoffs are obtained from experiments where fluorescent targets derived from the same RNA and labeled with either Cy3 or Cy5 are co-hybridized to the same microarray. For direct comparisons we performed two self-self direct hybridizations for each tissue. Thus, 6 replicate ratios for each spotted probe were generated: K1L/K2L, K1R/K2R, P1L/P2L, P1R/P2R, B1L/B2L, B1R/B2R, where K, P and B denote Kidney, Prostate and Breast RNAs, 1 and 2 denote either Cy3 or Cy5 dye labeling, and R and L denote the right and left array sets from each slide, respectively. This procedure yielded the experimental null distribution of the differential expression significance test since, by definition, there is no differential expression in the self-self dataset (see details in [22]). Concerning the accuracy of the gold-standard, it should be noted that this set of genes was selected by applying a statistical approach based on self-self hybridizations using a single RNA labeled with both Cy3 and Cy5. Therefore, any compression on the ratios due to systematic dye effects would also be present in the ratio cut-offs derived from these self-self experiments, thus cancelling out most of the possible bias in selecting the gold-standard set of genes. To derive a null distribution for the indirect comparisons, pseudo self-self ratios were reconstructed from replicate RefOligo, RefPool and One-Color data derived from the same RNA obtained from separate arrays. A total of 6 self-self ratios were thus generated for each RefOligo, RefPool and One-Color dataset. Next, we created 95% credibility intervals for both direct and indirect self-self log2-ratios [see Additional file 3]. These intervals were used to classify a given gene as differentially expressed if its replicate ratios in the pair-wise tissue comparisons were consistently (> 50%) outside the credibility interval thresholds. To be stringent we considered in this test only genes with more than 4 valid reconstructed ratios for the indirect dataset and only genes with all valid ratios for the direct dataset. Concordance and discordance among gene sets identified by direct and reconstructed ratios were represented as Venn diagrams.

## Authors' contributions

EMR and BRP conceived the initial idea, the experimental design, supervised the work, performed statistical analysis and wrote the manuscript. RZNV contributed with the experimental design and the development of R scripts for data processing and for the identification of differentially expressed genes using the HTself method. CE helped with nucleic acid isolation and performed all labeling and hybridization experiments. LMV articulated the collection of tumor samples along with detailed ethical guidelines. SVA contributed to the experimental design and to the writing of the manuscript and provided the facilities (wet lab and informatics) for execution of the experimental work. All authors read and approved the final manuscript.

## Supplementary Material

Additional file 1 MA-scatterplots from direct and reconstructed ratios. Click here for file

Additional file 3 Determination of intensity-dependent credibility intervals for differential gene expression in direct and indirect comparisons. Click here for file

Additional file 2 Correlation between direct and reconstructed ratios. Click here for file
