# Supplementary figures and images for: Evaluation of reference-based two-color methods for measurement of gene expression ratios using spotted cDNA microarrays
Source: BMC Genomics. 2006 Feb 24;7:35. doi: 10.1186/1471-2164-7-35 (PMC1402275; doi:10.1186/1471-2164-7-35)

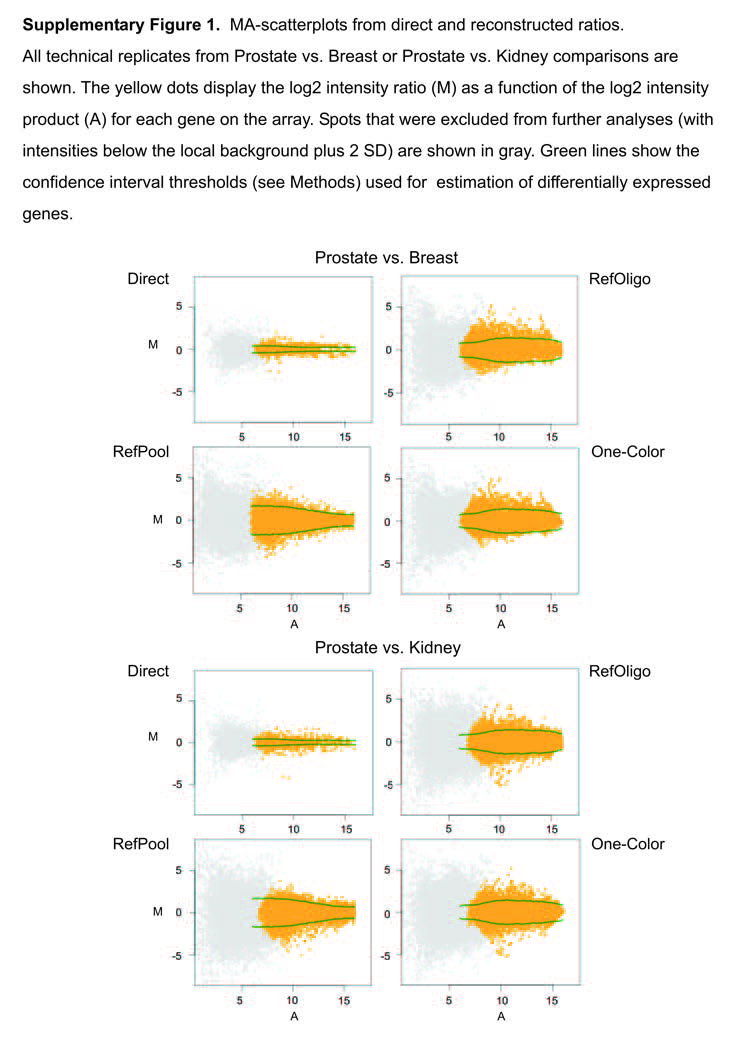

Supplement: Additional file 1 — MA-scatterplots from direct and reconstructed ratios. [file 1471-2164-7-35-S1.tiff]

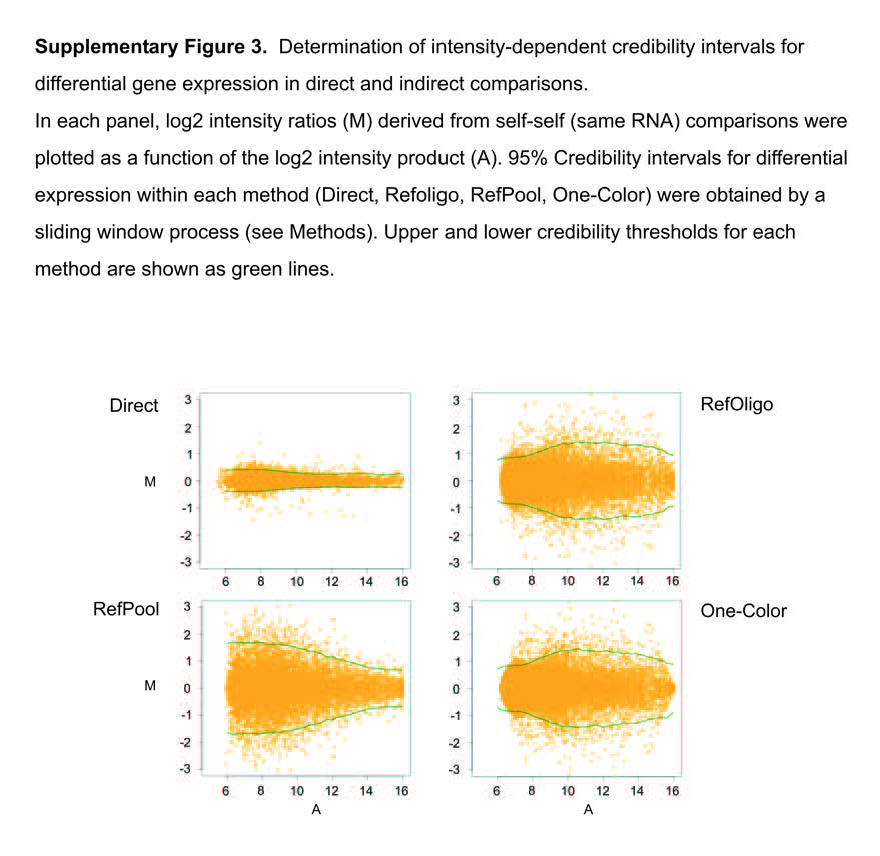

Supplement: Additional file 3 — Determination of intensity-dependent credibility intervals for differential gene expression in direct and indirect comparisons. [file 1471-2164-7-35-S3.tiff]

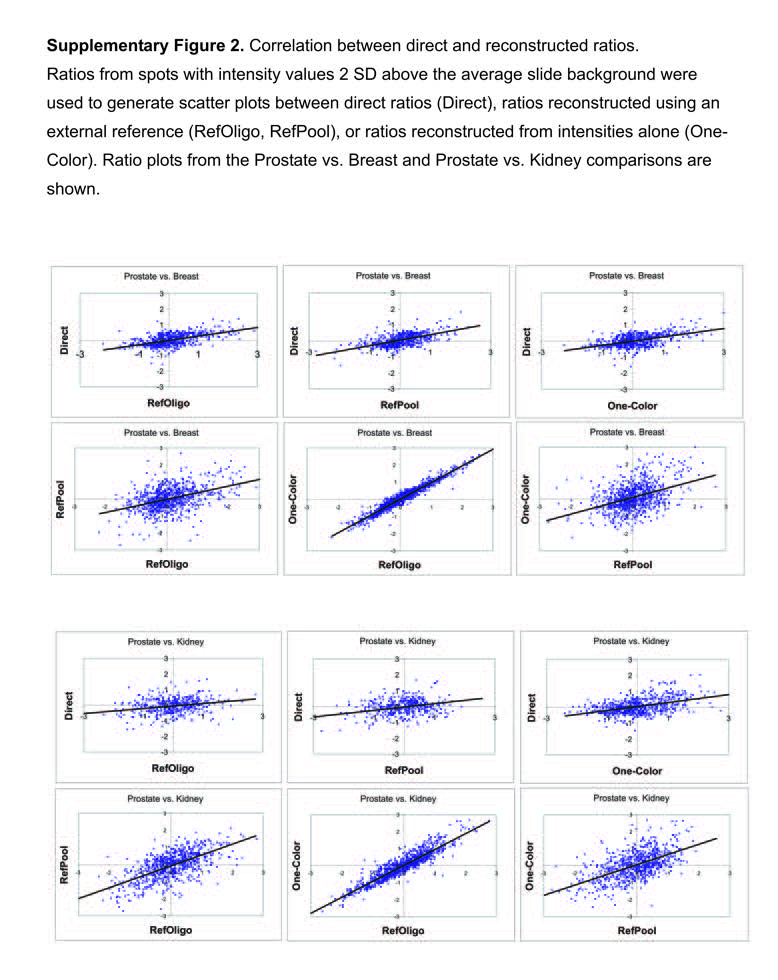

Supplement: Additional file 2 — Correlation between direct and reconstructed ratios. [file 1471-2164-7-35-S2.tiff]
